# The current state of the problem of in vitro gene pool preservation in poultry

**DOI:** 10.18699/VJ20.611

**Published:** 2020-03

**Authors:** Y.L. Silyukova, O.I. Stanishevskaya, N.V. Dementieva

**Affiliations:** Russian Research Institute of Farm Animal Genetics and Breeding – Branch of the L.K. Ernst Federal Science Center for Animal Husbandry, Pushkin, St. Petersburg, Russia; Russian Research Institute of Farm Animal Genetics and Breeding – Branch of the L.K. Ernst Federal Science Center for Animal Husbandry, Pushkin, St. Petersburg, Russia; Russian Research Institute of Farm Animal Genetics and Breeding – Branch of the L.K. Ernst Federal Science Center for Animal Husbandry, Pushkin, St. Petersburg, Russia

**Keywords:** semen, spermatozoa, marker proteins, cryopreservation, freeze-drying, poultry, gene pool preservation, cryobank, cryoresistance, семя, сперматозоид, маркерные белки, криоконсервация, лиофилизация, птицеводство, сохранение генетических ресурсов, криобанк, криорезистентность

## Abstract

This review presents the current progress in and approaches to in vitro conservation of reproductive cells of animals, including birds, such as cryopreservation and freeze-drying, as well as epigenetic conditions for restoring viable spermatozoa and female gametes after conservation. Cryopreservation is an effective way to preserve reproductive cells of various species of animals and birds. In vitro gene pool conservation is aimed primarily to the restoration of extinct breeds and populations and to the support of genetic diversity in populations prone to genetic drift. It is the combination of ex situ in vivo and ex situ in vitro methods that can form the basic principles of the strategy of animal genetic diversity preservation. Also, use of cryopreserved semen allows faster breeding in industrial poultry farming. Despite numerous advances in semen cryobiology, new methods that can more efficiently restore semen fertility after cryopreservation are being sought. The mechanisms underlying the effect of cryopreservation on the semen parameters of cocks are insufficiently understood. The review reflects the results of recent research in the field of cryopreservation of female and male germ cells, embryonic cells, the search for new ways in the field of genetic diversity in vitro (the development of new cryoprotective media and new conservation technologies: freeze-drying). Molecular aspects of cryopreservation and the mechanisms of cryopreservation influence on the epigenetic state of cells are highlighted. Data on the results of studies in the field of male reproductive cell lyophilization are presented. The freeze-drying of reproductive cells, as a technology for cheaper access to the genetic material of wild and domestic animals, compared to cryopreservation, attracts the attention of scientists in Japan, Israel, Egypt, Spain, and France. There is growing interest in the use of lyophilized semen in genetic engineering technologies. Methods of freeze-drying are developed taking into account the species of birds. Organizational and legal ways of solving the problems of in vitro conservation of genetic resources of farm animals, including birds, are proposed.

## Abstract (ru)

Настоящий обзор представляет современные достижения и подходы по сохранению репродуктивных клеток животных in vitro, такие как криоконсервация и лиофилизация, а также эпигенетические предпосылки для получения жизнеспособных сперматозоидов и женских гамет после реконсервации. Криоконсервация – эффективный путь сохранения репродуктивных клеток различных видов сельскохозяйственных животных, включая птиц. Метод сохранения генофонда in vitro через поддержание в криогенных условиях клеток или тканей в основном направлен на восстановление исчезнувших пород/популяций, на поддержание генетического разнообразия в популяциях, подверженных генетическому дрейфу. Именно сочетание методов ex situ in vivo и ex situ in vitro может сформировать основу эффективной стратегии сохранения генетического разнообразия животных. Кроме того, использование криконсервированного семени лучших представителей линии или породы позволяет ускорить прогресс селекции в промышленном птицеводстве. Несмотря на многочисленные достижения в области криобиологии половых клеток, продолжается поиск методов, обеспечивающих более эффективное восстановление жизнеспособности спермиев после криоконсервации. Механизмы, лежащие в основе влияния процедуры криоконсервации на параметры семени сельскохозяйственных птиц, полностью не изучены. В обзоре отражены результаты современных исследований в области проблематики криоконсервации женских и мужских половых клеток, эмбриональных клеток, поиска новых путей решения в области сохранения генетического разнообразия in vitro (разработка новых криопротекторных сред и новых технологий сохранения). Освещены молекулярно-генетические аспекты криоконсервации и механизмы влияния криоконсервации на эпигенетическое состояние клеток. Представлены данные по результатам исследований в области лиофильной сушки репродуктивных клеток самцов. Интерес к технологии лиофилизации семени как возможности более дешевого способа сохранения и транспортировки генетического материала диких и домашних животных, по сравнению с криоконсервацией, в мире стремительно растет; исследования ведутся в Японии, Израиле, Египте, Испании, Франции. Растет и интерес к использованию лиофилизированного семени в технологиях генной инженерии. Методы лиофильной сушки разрабатываются с учетом видовой принадлежности. В обзоре предложены также организационно-правовые пути решения проблемы сохранения генетических ресурсов сельскохозяйственных животных, включая птиц, in vitro.

## Introduction

The conservation of genetic resources of farm animals is a global challenge, and it attracts efforts of the world community. The Food and Agriculture Organization (FAO) of the United Nations and its specialized units coordinate these efforts (FAO, 2015).

Conservation programs for genetic resources include the following tasks: economyc (livestock husbandry support, response to changes in the environment, market needs, regulatory requirements, and the availability of imports and exports); social and cultural issues; conservation of biodiversity; and the maintenance of resources for academic or educational purposes, genetics, genomics, and adaptation to climate and other environmental changes.

The method of ex situ in vitro gene pool preservation through cryogenic maintenance (cryobank) of cells or tissues that can be used for breed/population restoration is recognized a necessary supplement to the in vivo method (FAO, 2015). It is the combination of ex situ in vivo and ex situ in vitro methods that can form grounds for an effective strategy for preserving animal genetic diversity.

Methods developed to freeze reproductive cells of male farm birds can be successfully applied to wild species to preserve their genetic diversity: red jungle chicken (Rakhaa et al., 2016), capercaillie (Kowalczyk et al., 2012), and pheasant (Saint Jaime et al., 2003). Due to the significant decrease in genetic diversity in pure lines of industrial crosses (Muir et al., 2008), the use of cryopreserved semen of the best representatives of a line or breed in artificial insemination under conditions of poultry industry expands the range of variation and accelerates breeding.

## Semen preservation

At present, cryopreservation of reproductive cells of males is the most important, practically the only method of preserving the gene pool of farm birds in vitro. Various protocols have been developed for the conservation of poultry semen, and the effectiveness of their use depends on many factors (Tselyutin, Tour, 2013; Thélie et al., 2019). The problem of reduced functional ability of the semen after a freeze-thaw cycle has not yet been resolved; the level of the fertilizing ability of thawed semen is not satisfactory. According to different authors, depending on the freezing methods, individual and breed characteristics of chickens, egg fertilization varies from 2 to 85 % (Blesbois et al., 2007; Long et al., 2010; Seigneurin, Blesbois, 2010; Çiftci, Aygün, 2018). The average level of fertilization with cryopreserved semen is low, usually less than 30 % (Fulton, 2006); however, some recent publications show 65 % average fertility of frozen-thawed semen (Silyukova et al., 2019). The reduced viability of embryos derived from cryopreserved semen caused by DNA fragmentation (Watson, 2000; Liptói, Hidas, 2006; Morris et al., 2012) also compromises the economic feasibility of its use for practical breeding purposes. Therefore, works aimed at the improvement of the composition of diluents for cryopreservation, the selection of cryoprotectant and freezing methods (in straws or pellets), the freezing protocols (low/fast), and so on are still under way (Thieu Ngoc Lan Phuong et al., 2014; Svoradová et al., 2017).

Most studies on cryopreservation of avian semen are conducted with mixed ejaculates from several males, although it is known that the genetic contributions of males differ due to the effect of selective fertilization (Sakharova, Popov, 2001), and males have different quality indicators of sperm after cryopreservation (Pleshanov et al., 2018, 2019). Therefore, there are concerns that the use of cryopreserved sperm may lead to an increase in inbreeding when using mixed sperm. In order to avoid this problem, when preserving rare and endangered breeds of chickens, it is necessary that the cryobank store individual ejaculates.

In Russia, studies in this direction are being conducted at the L.K. Ernst Federal Science Center for Animal Husbandry and its branch, the Russian Research Institute of Farm Animal Genetics and Breeding (Iolchiev et al., 2018; Mavrodina et al., 2018a, b; Pleshanov, Stanishevskaya, 2018; Stanishevskaya, Pleshanov, 2018a–c)

Technologies. Many scientific publications describe different protocols for cryopreservation of semen of domesticated and wild bird species. Technologies differ in the type of cryoprotectant, the method of packaging (straw, pellets, etc.), the speed of freezing and thawing (fast/slow) and temperature regimes. The effectiveness of the protocols can be assessed by analyzing the semen functional state in the laboratory (determination of sperm concentration, motility, morphology, and live/dead ratio) and assessing the fertilizing capacity of sperm in artificial insemination in vivo (Varadi et al., 2013; Thieu Ngoc Lan Phuong et al., 2014). It was found that high rates of avian semen freezing–thawing were preferable in terms of improving its survival in contrast to the protocol for mammalian semen (Shahverdi et al., 2015; Madeddu et al., 2016). There is significant individual, intrabreed, and intraspecific variation in bird semen cryostability, which demands that different cryopreservation strategies for different species and breeds be developed (Blesbois et al., 2007).

Methods of semen quality assessment. Semen cryopreservation is very important for ex situ management of avian genetic diversity, but the use of this method is limited due to the high variability of success rates. To calculate the number of sperm doses in the formation of the cryobank, it is necessary to predict the fertilizing capacity of cryopreserved semen. Unfortunately, in determining the effectiveness of their development, many researchers are limited only to the assessment of sperm motility. This test is not informative enough in terms of predicting the fertilizing capacity of semen.

A more effective prognosis for the fertilizing capacity of the semen is provided by assessment of morphological disorders including fluorescent staining of living and dead cells, flow cytometry, and evaluation of spermatozoon motility parameters using computer-assisted sperm analysis (CASA). The CASA system permits one to estimate the percentage of viable and morphologically normal cells (PVN), mass mobility (MMOT) and various parameters of movement, including the percentage of motile sperm (PMOT) and biophysical tests (resistance to osmotic stress (OSM), membrane permeability (FLUID)) (Blesbois et al., 2008; Svoradová et al., 2018). However, the set of these tests does not fully reflect the functionality of semen.

The functional capacity of thawed semen can be reliably determined in vitro by analyzing the interaction of the sperm with the inner layer of the perivitelline layer of the egg yolk (Robertson et al., 1997; Long et al., 2010). In evaluation of the functional state of spermatozoa in vitro in the laboratory, it is advisable to use the perivitelline membrane of egg yolk. The assessment is based on the number of hydrolysis points (spermatozoon penetrations) per unit area of the inner perivitelline layer (Robertson et al., 1997). This method, compared to traditional quality assessment, is more informative for predicting the fertilizing capacity of semen.

Preservation of female gametes. By now, no method has been developed to preserve germ cells of female birds. The presence of large amounts of yolk in the eggs of birds hampers the use of existing cryopreservation methods (Fulton, 2006). This is a serious issue in the preservation of a breed/population, as it cannot be fully preserved without the genetic contribution of individuals of both sexes: there is a loss of maternal hereditary material, including the mitochondrial genome. Currently existing methods of preservation of reproductive cells of birds (semen) allow restoration of endangered breeds/ populations only by grading.

A relatively new technology is the transplantation of cryopreserved gonad cells from neonatal chickens to adult recipients for reproduction of donor offspring. This method of transplantation can contribute to the conservation of endangered bird species and maintain their genetic variability (Benesova, Trefil, 2016). Cryopreservation of ovarian tissue is actually the only effective way to preserve in vitro female germ plasma in birds. A method of vitrification of donor ovarian tissues in straw is proposed. Ovarian tissue fragments from 1-week old females are transferred to a metal rod, then vitrified in liquid nitrogen using special media. By this method, the ovarian tissue of birds can be stored and transported. Successful transplant survival was demonstrated by Liu et al. (2012). In addition, ovarian transplantation can be used for research in genetics and developmental biology (Song, Silversides, 2007; Liptoi et al., 2013). Since this technique involves significant surgery and requires the use of immunosuppressants, today it appears to be costly and technologically challenging in routine implementation.

Preservation of embryonic cells. Chicken primary germ cells (PGCs) can be isolated and cultured in vitro. PGCs act as a valuable source material for cellular genetic engineering, germ plasm production, and genetic conservation of species and populations (Kino et al., 1997). Indeed, bird PGCs can be reproduced in culture and conserved without irreversible alteration of their biological properties (van der Lavoir et al., 2006; Nandi et al., 2016; Tonus et al., 2016). Two main methods are commonly used for cryopreservation of PGCs in birds: slow freezing (SLF) and ultrafast freezing (Vitrif) (Tonus et al., 2017). These cells can be used to repair gonadal tissues with germ cells of the donor line. This method is not currently applicable for the preservation of entire embryos (Fulton, 2006). Both methods require further research, but we can now definitely state that in the future this approach to in vitro preservation of cells will provide grounds for the development of a practical gene bank and systematic genomic bank for birds.

## Genetics of cryostability of reproductive cells

It has been established that semen cryostability is a genetically determined trait (Pleshanov et al., 2019), but the mechanisms of the effect of cryopreservation on the epigenetic state of cells have not yet been fully investigated. Semen freezing–thawing can injure genes, including SNORD116/PWSAS and UBE3A, associated with fertility (Valcarce et al., 2013).

The study of changes in boar semen after cryopreservation revealed differences in 41 proteins (Chen et al., 2014). Proteins SOD1, TPI1, ODF2, and AKAP3 have been proposed as markers affecting semen resistance to freezing. In Gallus gallus domesticus, ontology genes were found for SOD1, TPI1, and ODF2. Consider these genes in more detail. The protein encoded by the SOD1 gene (Superoxide dismutase 1) binds copper and zinc ions and promotes the breakdown of superoxide radicals into molecular oxygen and hydrogen peroxide (Bogle et al., 2017; Wu, 2019). Another isozyme of this protein is found in mitochondria, and its functions have not yet been studied. The enzyme TPI1 (Triosephosphate isomerase 1), which consists of two identical proteins, catalyzes the isomerization of glyceraldehyde 3-phosphate (G3P) and dihydroxyacetone phosphate (DHAP) in glycolysis and gluconeogenesis (Chen et al., 2014).

It has been found that HSP90 (Heat shock protein 90) proteins are associated with sperm motility, and their pool decreases significantly after freezing–thawing (Huang et al., 2009).

Significant protein changes in human sperm before and after cryopreservation were detected by Wang et al. (2014): mitochondrial matrix proteins ACO2 (Aconitase 2) and OXCT1 (3-oxoacid CoA-transferase 1); filamentous protein TEKT1 (Tektin 1), which is necessary for the formation of ciliary and flagellar microtubules; glycolytic enzyme ENO1 (Enolase 1), the intermediate filament protein vimentin; and the amino acid tyrosine. These molecules are associated with sperm motility, viability, and acrosome integrity (Wang et al., 2014).

As a result of semen freeze–thawing, the amounts of antioxidant proteins such as SOD1, PRDX6 (Peroxiredoxin 6), TXNDC2 (Thioredoxin containing domain 2), GSTM3 (Glutathione- S-transferase mu 3), membrane proteins CYB5R2 (Cytochrome b5 reductase 2), pellucid zone proteins ZPBP1 and ZPBP2 (Zona pellucida binding protein), acrosomal proteins ACRBP (Acrosin binding protein), and SPACA3 (Sperm acrosome associated 3) were found to decrease. Simultaneously, the amounts of other proteins whose accumulation is observed in cells under stress – ANX1, ANX3, and ANX4 (Annexin A); clusterin (CLU Clusterin); importin-1b (KPNB1); Karyopherin subunit beta 1, HIST1H4A (Histone cluster 1 H4 family member a); TUBA1A (Tubulin alpha 1a); and SPAG17 (Sperm associated antigen 17) increased (Bogle et al., 2017).

A study of the effect of cryopreservation on Gallus gallus domesticus spermatozoa has shown an increase in the amounts of 36 proteins and a decrease in 19 proteins after thawing. These proteins are linked to spermatozoa metabolism (Cheng et al., 2015). Proteins such as ACRBP, FN1 (Fibronectin 1), HSP90AA1 (Heat shock protein 90), and VDAC2 (Voltage dependent anion channel 2) are biomarkers that predict tolerance of cryopreservation in boar semen (Vilagran et al., 2015, 2016).

During fertilization, spermatozoa deliver the paternal mRNA to the egg and thus play an important role in the early development of the embryo. During freezing, transcripts and mRNA–protein interactions in spermatozoa may be lost, which may affect embryo development (Valcarce et al., 2013). Correlations between sperm mRNA and early embryo development in humans and some animals were identified in (Hezavehei et al., 2018). Studies by Valcarce et al. (2013) showed a decrease in the expression of the PRM1, PRM2, PEG1/MEST, and ADD1 genes associated with human sperm fertility after cryopreservation. Some studies confirmed changes in transcripts of some proteins and micro-RNA. Attempts are being made to explain some epigenetic modifications that may occur in spermatozoa during freezing (Hezavehei et al., 2018).

Cryopreservation of semen is a very important method of assisted reproduction, but the freezing–thawing procedure is harmful, because it leads to a decrease in the motility and viability of spermatozoa, premature capacitation, and, as a consequence, poorer effectiveness of artificial fertilization. Therefore, the addition of some proteins normalizes the process of condensation and accelerates fertilization in vitro. For example, use of TrxA-FNIIx4-His6 is a promising biotechnological approach for cryopreservation of ram semen and maintenance of spermatozoon viability (Ledesma et al., 2019).

In addition to preserving genetic material by creating a sperm bank, it is possible to create a cryobank of embryos. In cattle, the effect of resveratrol on embryos after cryopreservation was evaluated. Its effects on mitochondrial function preservation, DNA integrity, SIRT1 (Sirtuin 1) expression and embryo development ability have been studied. Embryo survival was significantly improved when embryos were incubated in a medium containing 0.5 μM of resveratrol after thawing. Besides, SIRT1 expression and cell-free mtDNA content in the medium were higher in the case of embryos treated with resveratrol. It should be noted that slow freezing affects mitochondrial integrity and function in blastocysts (Hayashi et al., 2019). It is important to improve in vitro maturation (IVM) conditions for immature oocytes after cryopreservation, especially if a limited number of oocytes are collected from specific donors. Culture systems with fresh oocytes significantly accelerate the meiotic development of vitrified oocytes and significantly increase the rate of blastocyst formation after parthenogenetic activation and transfer of somatic cell nuclei (Jia et al., 2019).

The understanding of the molecular mechanisms that determine epigenetic processes occurring in reproductive cells during freezing–thawing will improve the effectiveness of the technologies used to preserve species, breeds, and populations of rare and endangered animals and birds.

## Lyophilization

The preservation of semen by freeze-drying is an innovative method. The advantages of lyophilized semen are that it can be (1) stored at 4 °C for a long time and (2) stored and transported at room temperature without the use of liquid nitrogen or dry ice as cooling agents.

It is expected that sperm lyophilization, rather than cryopreservation, can become a new simple method of preserving genetic resources and be used, among other things, to produce transgenic animals (Kaneko, 2012). The state of research in the field of freeze-drying of wild and domestic animal semen indicates an increasing interest in this method of preserving genetic resources. Methods of lyophilization in relation to microorganisms and plant cells have been developed and successfully applied. Interest in the lyophilization of reproductive cells, as a possibility of a cheaper way to preserve and transport (including space) genetic material of wild and domestic animals, compared with cryopreservation, is growing rapidly in the world; research is underway in Japan, Israel, Egypt, Spain, and France. Methods of lyophilic drying are developed with regard to species features. Promising results have been achieved in mice, rats, hamsters, cattle, sheep, rabbits, chimpanzees, giraffes, jaguars, etc., but it is too early to talk about the problem as solved, since the functional characteristics of sperm are not fully preserved (Hopshi et al., 1994; Foote, 2002; Liu et al., 2004; Kawase et al., 2005; Li et al., 2009; Gil et al., 2014; Kaneko et al., 2014; Shahba et al., 2016; Wakayama et al., 2017; Arav et al., 2018). The main issues are associated with damage of the motility apparatus of spermatozoa, membranes, and DNA. As for birds, including poultry, research on the freeze-drying of their semen has not been carried out, at least not published.

## Problems of cryopreservation

Cryopreservation triggers damage processes not only at the mechanical level of membrane damage, but also chemical and physical processes of denaturation of proteins and lipids of membrane bilayers. These processes result in sublethal freezing and the launch of cryocapacitation, generation of reactive oxygen species, and aberrations in sperm proteins, lipids, and sugars (Pini et al., 2018).

It is well known that the tolerance of cold shock and cryostability in spermatozoa of different livestock species, including farm birds, varies greatly. Cryopreserved semen of any animal species has reduced fertility compared to fresh sperm. The causes of fertility loss include the susceptibility to cold shock, cooling rate, diluent composition, and osmotic stress. There are also factors that affect the functional state of frozen/ thawed spermatozoa: membrane stability, oxidative damage, membrane receptor integrity, and nucleus structure (Watson, 2000; Iolchiev et al., 2018). In the course of cryopreservation and thawing, sperm can experience both irreversible damage, expressed as the absence of motility and various morphological disorders, and reversible, associated mainly with a temporary injury of the structure and membrane permeability disturbance.

It is believed that the high content of intracellular protein together with osmotic “shrinkage” of the sperm membrane associated with the formation of extracellular ice leads to intracellular vitrification of sperm during cooling. At high cooling rates, sperm damage is the result of osmotic imbalance occurring during thawing, rather than intracellular ice formation during freezing. Osmotic imbalance occurs at high cooling rates due to limited diffusion of ice crystallization in the extracellular fluid; that is, the amount of ice formed during cooling is less than expected from the phase equilibrium diagram (Morris et al., 2012).

A significant interbreeding variability in the cryostability of cock semen, estimated by the activity of thawed semen, is known: the coefficient of variation (Cv ) can be up to 23–25 % (Pleshanov et al., 2018; Stanishevskaya, Pleshanov, 2018a). A greater individual variability in cock spermatozoon activity in the freeze/thaw cycle has been shown in (Pleshanov et al., 2017; Pleshanov, Stanishevskaya, 2018; Stanishevskaya, Pleshanov, 2018a, b). The coefficient of variation (Cv ) of spermatozoon activity was 6.1 % in native sperm and 19.5 % in frozen/thawed, which points to a broad norm of sperm response to low temperatures.

The generally accepted parameters of ejaculate selection for cryopreservation are volume, concentration, and spermatozoon motility. These criteria do not provide a complete prediction of the degree of reproductive cell, cryotolerance, which is largely due to the state of the membranes, as the membranes are first to be damaged in the freezing–thawing process.

One of the ways to assess the degree of cryopreservation of spermatozoon membranes is staining with Sperm VitalStain dye (Nidacon International AB, Sweden), which allows assessment of the degree of cryopreservation by changing the color of damaged cells (Pleshnov, Stanishevskaya, 2018). Lipid fractions of membranes, such as glycolipids, phospholipids, sterols, cholesterol, the cholesterol/phospholipids ratio, etc., affect the state of cell membranes; their permeability, microviscosity, and fluidity; molecular mobility of lipids in the membrane; the process of capacitation, the interaction of egg and spermatozoon membranes; and the result of fertilization (Blesbois et al., 2005; Ahmed et al., 2014; Eubaid et al., 2015; Partyka et al., 2016; Pleshanov et al., 2017).

Recent studies of sperm cryostability have established the effect of the amino acid profiles of seminal plasma in different breeds of chickens on DNA fragmentation (Santiago-Moreno et al., 2019). The composition of intracellular spermatozoon protein has been found to be associated with indicators of osmotic imbalance after thawing (Morris et al., 2012). The results of these studies open up new aspects of sperm cryobiology, which is a prerequisite for the development of new technologies for semen preservation, including vitrification and lyophilization.

Problems of early embryonic mortality. It is well known that the use of frozen/thawed semen reduces not only the percentage of their fertility but also the viability of embryos. The mortality rate of embryos in the early stages of development can reach 8–17 % (Stanishevskaya, Pleshanov, 2018c). This research area is insufficiently studied, as it is technically difficult to investigate the causes of arrested development, since signs of early embryonic death are not determined. DNA damage is probably a major cause of early embryonic mortality caused by functional damage to sperm nuclear structures (Watson, 2000; Liptói, Hidas, 2006). In addition, the influence of toxic endo/exocellular cryoprotectants used in sperm freezing and their concentrations, which may also cause embryo death at an early stage of development, should not be ruled out (Mosca et al., 2019).

Thus, the genetic diversity of the preserved material is reduced at different stages of postsingamy due to the elimination of individuals with reduced cryoresistance of reproductive cells.

Cryoprotectants. A necessary condition for successful cryopreservation of reproductive cells is the use of cryoprotectants. Cryoprotectants acting inside cells penetrate into cells and prevent the formation of intracellular ice, but at high concentrations they exert a damaging effect. Exocellular cryoprotectants act outside the sperm in the extracellular space and protect cells by dehydrating the intracellular space and limiting the action of osmotic shock during thawing.

Glycerol, one of the best known cryoprotectants, is the most effective and less toxic to cock sperm, but unfortunately has a contraceptive effect after insemination of hens and requires removal before insemination. The most widely used penetrating cryoprotectants are dimethylsulfoxide, dimethylacetamide, dimethylformamide and ethyleneglycol. Semen samples can be thawed without further processing, and high fertility levels are obtained with these substances depending on the cooling rate and the type of semen packaging (Santiago-Moreno et al., 2011). Nonpenetrating cryoprotectants, also known as osmoprotectants, are low-molecular-weight hydrophilic nontoxic molecules that stabilize internal solutes under osmotic stress in cells. These cryoprotectants are often used in combination with penetrators (Benesona, Trefil, 2016; Mosca et al., 2016; Svoradová et al., 2017).

Recent approaches to the development of cryoprotectants of fundamentally different actions are based on antifreeze glycoproteins (AFGP) and antifreeze proteins (AFP), found in the blood and tissues of poikilothermic organisms living in freezing environments (insects and marine fish). The obtained substances inhibit the growth of ice crystals in a noncolligative manner. The use of AFP opens up a promising direction for cryopreservation of living tissues and cells. The efficacy of some fish AFP or fish AFGP against hypothermic damage has been reported while preserving swine and cattle oocytes, whole rat liver, and model membranes. To preserve sperm, attempts have been made to develop cryopreservation methods with the addition of fish AFP in different species with different efficiencies. AFP and AFGP of marine fish have recently been found to improve buffalo sperm cryopreservation results (Qadeer et al., 2016).

There are studies on the use of recombinant AFP-based Dendroides canadensis (DAFP) larvae for cryopreservation. Addition of DAFP to the diluent protects buffalo (Bubalus bubalis) semen during freezing–thawing and increases the fertility of cryopreserved semen (Qadeer et al., 2016).

## Cryobanks and their contribution to the conservation of genetic resources

Collections of genetic banks are of paramount importance in preventing the extinction of breeds due to extreme genetic conditions, such as small breed/population size and high incidence of genetic defects as a result of intensive breeding and genetic drift. Stored material from animals that do not carry undesired or lethal mutations can be used to reduce the frequency of defects to an acceptable level.

Biobanks are a ready-to-use source of genetically diverse and specialized DNA. Conserved materials are used in studying genetic diversity, genomic associations, gene functions, and other issues. Importantly, over time, genetic banks can provide samples from different generations, which contributes to the accuracy of genomic selection. These advantages will be easier to implement if the information is catalogued taking into account the phenotype and genotype and the stored samples have genomic certificates (Wildt, 2000; Comizzoli, 2015).

The problem of in vitro preservation of genetic diversity, including farm birds, is being solved in many countries of the world. One of the advantages of preserving genetic diversity in vitro in cryobanks is the economic component (Woelders, 2006; Santiago-Moreno et al., 2011; Silversides et al., 2013; FAO, 2015).

Recently, a new approach to interaction between organizations preserving the gene pool ex situ in vivo and ex situ in vitro has been developed. The goal of the genetic bank is not only to obtain and preserve reserve biological material but also to closely cooperate with collections in live breeding to expand genetic diversity while preserving ex situ in vivo.

Genetic banks for the conservation of farm birds can take into account the experience of the European Union, the European Federation of Animal Science (EAAP), and FAO, which have established European and international consultative forums to discuss and take practical measures for the conservation of genetic resources worldwide. However, the implementation of this idea is a complex process, which requires interdisciplinary cooperation and clear definition of goals (Mara et al., 2013).

The legislation of the Russian Federation provides for a regulatory framework (Strategy) for the conservation of rare and endangered species of animals, plants and fungi (Order No. 212-r of the Government of the Russian Federation of 17.02.2014), including preservation in vitro. As for the problem of preserving the genetic diversity of farm animals and birds, the Federal law No. 123-FZ “On livestock breeding” of 03.08.1995 does not provide for such regulation. It is necessary to develop and adopt a law and by-laws that would determine the legal status of genetic cryobanks in the overall system of genetic resource conservation.

## Conflict of interest

The authors declare no conflict of interest.

## References

Ahmed M., Sattar A., Iqbal S., Shahzad Q., Zahid Tahir M., Hammad
Fayyaz M., Ahmad M. Cholesterol loaded cyclodextrin improves
sperm survival in tris-based extender with less egg yolk used
in buffalo bulls semen. Thai J. Vet. Med. Suppl. 2014;44:129-130.

Arav A., Idda A., Nieddu S., Natan Y., Ledda S. High post-thaw survival
of ram sperm after partial freeze-drying. J. Assist. Reprod. Genet.
2018;35(7):1149-1155. DOI 10.1007/s10815-018-1145-1.

Benesova B., Trefil P. Possibilities for preserving genetic resources
in birds. World’s Poult. Sci. J. 2016;72(3):628-641. DOI 10.1017/
S0043933916000489.

Blesbois E., Grasseau I., Segineurin F. Membrane fluidity and the ability
of domestic bird spermatozoa to survive cryopreservation. Reproduction.
2005;129(3):371-378. DOI 10.1530/rep.1.00454.

Blesbois E., Grasseau I., Seigneurin F., Mignon-Grasteau S., Saint
Jalme M., Mialon-Richard M.M. Predictors of success of semen
cryopreservation in chickens. Theriogenology. 2008;69(2):252-261.
DOI 10.1016/j.theriogenology.2007.09.019.

Blesbois E., Seigneurin F., Grasseau I., Limouzin C., Besnard J., Gourichon
D., Coquerelle G., Rault P., Tixier-Boichard M. Semen cryopreservation
for ex situ management of genetic diversity in chicken:
creation of the French avian cryobank. Poult. Sci. 2007;86(3):555-
564. DOI 10.1093/ps/86.3.555-564.

Bogle O.A., Kumar K., Attardo-Parrinello C., Lewis S.E., Estanyol
J.M., Ballescà J.L., Oliva R. Identification of protein changes
in human spermatozoa throughout the cryopreservation process.
Andrology.
2017;5:10-22. DOI 10.1111/andr.12279.

Chen X., Zhu H., Hu C., Hao H., Zhang J., Li K., Zhao X., Qin T.,
Zhao K., Zhu H., Wang D. Identification of differentially expressed
proteins in fresh and frozen-thawed boar spermatozoa by iTRAQcoupled
2D LC-MS/MS. Reproduction. 2014;147(3):321-330. DOI
10.1530/REP-13-0313.

Cheng C.Y., Chen P.R., Chen C.J., Wang S.H., Chen C.F., Lee Y.P.,
Huang S.Y. Differential protein expression in chicken spermatozoa
before and after freezing-thawing treatment. Anim. Reprod. Sci.
2015;152:99-107. DOI 10.1016.anireprosci.2014.11.011.

Çiftci Y., Aygün A. Poultry semen cryopreservation technologies.
World’s Poult. Sci. J. 2018;74(4):699-710. DOI 10.1017/s0043933
918000673.

Comizzoli P. Biobanking efforts and new advances in male fertility
preservation for rare and endangered species. Asian J. Androl. 2015;
17:640-645. DOI 10.4103/1008-682X.153849.

Eubaid H.J., Al-Haidary B.A., Tawfiq L.J. Possible role for cholesterol
in human seminal fluidin relations to other semen parameters & fertility.
J. Babylon Univ./Pure Appl. Sci. 2015;23(2):654-659.

FAO. 2015. The Second Report on the State of the World’s Animal
Genetic Resources for Food and Agriculture. Ed. by B.D. Scherf,
D. Pilling. FAO Commission on Genetic Resources for Food and
Agriculture Assessments. Rome, 2015. Available at: http://www.fao.
org/3/a-i4787e/index.html

Foote R. The history of artificial insemination; selected notes and notables.
J. Anim. Sci. 2002;80:1-10.

Fulton J.E. Avian genetic stock preservation: an industry perspective.
Poult. Sci. 2006;85:227-231.

Gil L., Olaciregui M., Luno V., Malo C., González N., Martínez F. Current
status of freeze-drying technology to preserve domestic animals
sperm. Reprod. Domest. Anim. 2014;49(4):72-81. DOI 10.1111/rda.
12396.

Hayashi T., Kansaku K., Abe T., Ueda S., Iwata H. Effects of resveratrol
treatment on mitochondria and subsequent embryonic development
of bovine blastocysts cryopreserved by slow freezing. Anim. Sci. J.
2019;90(7):849-856. DOI 10.1111/asj.13219.

Hezavehei M., Sharafi M., Kouchesfahani H.M., Henkel R., Agarwal
A., Esmaeili V., Shahverdi A. Sperm cryopreservation: a review
on current molecular cryobiology and advanced approaches. Reprod.
BioMed. Online. 2018;37(3):327-339. DOI 10.1016/j.rbmo.
2018.05.012.

Hopshi K., Yanagida K., Katayose H., Yazawa H. Pronuclear formation
and cleavage of mammalian eggs after microsurgical injection of
freeze-dried sperm nuclei. Zigote. 1994;2:237-242. DOI 10.1017/
S0967199400002033.

Huang S.Y., Pribenszky C., Kuo Y.H., Teng S.H., Chen Y.H.,
Chung M.T., Chiu Y.F. Hydrostatic pressure pre-treatment affects
the protein profile of boar sperm before and after freezing-thawing.
Anim. Reprod. Sci. 2009;112:136-149. DOI 10.1016/j.anireprosci.
2008.04.016.

Iolchiev B.S., Bagirov V.A., Zhilinsky M.A., Volkova N.A., Zinovieva
N.A. Change of biological parameters of poultry semen at
cryopreservation. Sel’skohozyaystvennaya Biologiya = Agricultural
Biology. 2018;6(53):1230-1237. DOI 10.15389/agrobiology.2018.6.
1230eng.

Jia B.Y., Xiang D.C., Zhang B., Quan G.B., Shao Q.Y., Hong Q.H.,
Wu G.Q. Quality of vitrified porcine immature oocytes is improved
by coculture with fresh oocytes during in vitro maturation. Mol. Reprod.
Dev. 2019;86:1615-1627. DOI 10.1002/mrd.23249.

Kaneko T. New possibilities of sperm freeze-drying. J. Fert. in Vitro.
2012;2:5. DOI 10.4172/2165-7491.1000e119.

Kaneko T., Ito H., Sakamoto H., Onuma M., Inoue-Murayama M.
Sperm preservation by freeze-drying for the conservation of wild
animals. PLoS One. 2014;9(11):e113381. DOI 10.1371/journal.
pone.0113381.g001.

Kawase Y., Araya H., Kamada N., Jishage K., Suzuki H. Possibility
of long-term preservation of freeze-dried mouse spermatozoa.
Biol. Reprod. 2005;72(3):568-573. DOI 10.1095/biolreprod.104.
035279.

Kino K., Pain B., Leibo S.P., Cochran M., Clark M.E., Etches R.J.
Production of chicken chimeras from injection of frozen-thawed
blastodermal cells. Poult. Sci. 1997;76(5):753-760. DOI 10.1093/
ps/76.5.753.

Kowalczyk A., Lukaszewicz E.T., Rzońca Z. Successful preservation of
capercaillie (Tetrao urogallus L.) semen in liquid and frozen states.
Theriogenology. 2012;77:899-907. DOI 10.1016/j.theriogenology.
2011.09.015.

Ledesma A., Zalazar L., Buchelly Imbachi F., Pastore J.I., Brown P.,
Eddy E.M., Hozbor F., Cesari A. Recombinant peptide reverses
cryo-capacitation in ram sperm and improves in vitro fertilization.
Anim. Reprod. Sci. 2019;207:61-72. DOI 10.1016/j.anireprosci.
2019.05.016.

Li M.-W., Willis B.J., Griffey S.M., Spearow J.L., Lloyd K.C. Assessment
of three generations of mice derived by ICSI using
freeze-dried sperm. Zygote. 2009;17(03):239-251. DOI 10.1017/
s0967199409005292.

Liptói K., Hidas A. Investigation of possible genetic background of
early embryonic mortality in poultry. World’s Poult. Sci. J. 2006;
62(02):326-337. DOI 10.1079/wps2005101.

Liptoi K., Horvath G., Gal J., Varadi E., Barna J. Preliminary results of
the application of gonadal tissue transfer in various chicken breeds
in the poultry gene conservation. Anim. Reprod. Sci. 2013;141(1-2):
86-89. DOI 10.1016/j.anireprosci.2013.06.016.

Liu J., Cheng K.M., Silversides F.G. Novel needle-in-straw vitrification
can effectively preserve the follicle morphology, viability, and
vascularization of ovarian tissue in Japanese quail (Coturnix japonica).
Anim. Reprod. Sci. 2012;134(3-4):197-202. DOI 10.1016/
j.anireprosci.2012.08.002.

Liu J.-L., Kusakabe H., Chang C.-C., Suzuki H., Schmidt D.W., Julian
M., Pfeffer R., Bormann C.L., Tian X.C., Yanagimachi R.,
Yang X. Freeze-dried sperm fertilization leads to full-term development
in rabbits. Biol. Reprod. 2004;70(6):1776-1781. DOI 10.1095/
biolreprod.103.025957.

Long J.A., Bongalhardo D.C., Pelaez J., Saxena S., Settar P., O’Sullivan
N.P., Fulton J.E. Rooster semen cryopreservation: effect of
pedigree line and male age on post-thaw sperm function. Poult. Sci.
2010;89(5):966-973. DOI 10.3382/ps.2009-00227.

Madeddu M., Mosca F., Abdel Sayed A., Zaniboni L., Mangiagalli
M.G., Colombo E., Cerolini S. Effect of cooling rate on the
survival of cryopreserved rooster sperm: comparison of different
distances in the vapor above the surface of the liquid nitrogen.
Anim. Reprod. Sci. 2016;171:58-64. DOI 10.1016/j.anireprosci.2016.
05.014.

Mara L., Casu S., Carta A., Dattena M. Cryobanking of farm animal
gametes and embryos as a means of conserving livestock genetics.
Anim. Reprod. Sci. 2013;38(1-2):25-38. DOI 10.1016/j.anireprosci.
2013.02.006.

Mavrodina T., Stanishevskaya O., Cherepanov S., Silyukova Y. Influence
of sperm quality (cryopreserved and native) on the duration
of spermatozoa storage in reproductive tracts of turkeys.
Anim. Reprod. Sci. 2018a;194:e13. DOI 10.1016/j.anireprosci.2018.
04.034.

Mavrodina T., Stanishevskaya O., Cherepanov S., Silyukova Y. Influence
of osmolality of the media for dilution and cryopreservation of
turkey toms’ sperm on fertilization ability of thawed sperm. Reprod.
Domest. Anim. 2018b;53(2):164.

Morris G.J., Acton E., Murray B.J., Fonseca F. Freezing injury: the
special case of the sperm cell. Cryobiology. 2012;64:71-80. DOI
10.1016/j.cryobiol.2011.12.002.

Mosca F., Madeddu M., Abdel Sayed A., Zaniboni L., Iaffaldano N.,
Cerolini S. Combined effect of permeant and non-permeant cryoprotectants
on the quality of frozen/thawed chicken sperm. Cryobiology.
2016;73(3):343-347. DOI 10.1016/j.cryobiol.2016.10.001.

Mosca F., Zaniboni L., Abdel Sayed A., Madeddu M., Iaffaldano N.,
Cerolini S. Effect of dimethylacetamide and N-methylacetamide on
the quality and fertility of frozen/thawed chicken semen. Poult. Sci.
2019;98(11):6071-6077. DOI 10.3382/ps/pez303.

Muir W., Wong G.-K., Zhang Y., Wang J., Groenen M., Crooijmans R.,
Megens H.-J., Zhang H., Okimoto R., Vereijken A., Jungerius A.,
Albers
G., Lawley C., Delany M., MacEachern S., Cheng H.
Genome-
wide assessment of worldwide chicken SNP genetic diversity
indicates significant absence of rare alleles in commercial
breeds. Proc. Natl. Acad. Sci. USA. 2008;105(45):17312-17317.
DOI 10.1073/pnas.0806569105.

Nandi S., Whyte J., Taylor L., Sherman A., Nair V., Kaiser P.,
McGrew
M.J. Cryopreservation of specialized chicken lines using
cultured primordial germ cells. Poult. Sci. 2016;95:1905-1911.

Partyka A., Bonarska-Kujawa D., Sporniak M., Strojecki M., Niżański
W. Modification of membrane cholesterol and its impact on
frozen-thawed chicken sperm characteristics. Zygote. 2016;24(5):
714-723.

Pini T., Leahy T., Graaf S. Sublethal sperm freezing damage: manifestations
and solutions. Theriogenology. 2018;118:172-181. DOI
10.1016/j.theriogenology.2018.06.006.

Pleshanov N., Cherepanov S., Stanishevskaya O. Chicken sperm cryopreservation
as a tool of maintenance genetic diversity in small
scale populations (Proc. of the XVth Eur. Poultry Conf. Dubrovnik).
World’s Poult. Sci. J. 2018:445.

Pleshanov N., Stanishevskaya O. Evaluation of the cocks spermatozoa
membranes’ damaging during cryopreservation with use of Sperm
VitalStain colorant. Reprod. Domest. Anim. 2018;53(S2):183.

Pleshanov N., Stanishevskaya O., Silyukova Y. Fertilizing ability of the
cryopreserved cock sperm depending on cholesterol levels and cholesterol
impact on the degree of damaging of the membrane structures
in spermatozoa. Genetika i Razvedenie Zhivotnyh = Genetics
and Breeding of Animals. 2017;3:34-40. (in Russian)

Pleshanov N., Stanishevskaya O., Silyukova Y. Inheritance of cock’s
sperm cryostability. Reprod. Domest. Anim. 2019;54(3):133.

Qadeer S., Khan M., Shahzad Q., Azam A., Ansari M., Rakha B.,
Ejaz R., Husna A., Duman J., Akhter S. Efficiency of beetle (Dendroides
canadensis) recombinant antifreeze protein for buffalo semen
freezability and fertility. Theriogenology. 2016;86(7):1662-
1669. DOI 10.1016/j.theriogenology.2016.05.028.

Rakhaa B., Ansarib M., Akhterc S., Hussaina I., Blesboisd E. Cryopreservation
of Indian red jungle fowl (Gallus gallusmurghi) semen.
Anim. Reprod. Sci. 2016;174:45-55. DOI 10.1016/j.anireprosci.
2016.09.004.

Robertson L., Brown H., Staines H., Wishart G. Characterization and
application of an avian in vitro spermatozoa-egg interaction assay
using the inner perivitelline layer from laid chicken eggs. J. Reprod.
Fertility. 1997;110(2):205-211.

Saint Jalme M., Lecoq R., Seigneurin F., Blesbois E., Plouzeau E.
Cryopreservation of semen from endangered pheasants: the first step
towards a cryobank for endangered avian species. Theriogenology.
2003;59(3-4):875-888. DOI 10.1016/s0093-691x(02)01153-6.

Sakharova S.A., Popov I.I. Improvement of chicken gene pool conservation
methods. Ptahivnictvo = Poultry Farming. 2001;51:135-141.
(in Ukrainian)

Santiago-Moreno J., Bernal B., Pérez-Cerezales S., Castaño C., Toledano-
Díaz A., Esteso M.C., Gutiérrez-Adán A., López-Sebastián A.,
Gil M.G., Woelders H., Blesbois E. Seminal plasma amino acid profile
in different breeds of chicken: role of seminal plasma on sperm
cryoresistance. PLoS One. 2019;14(1):e0209910. DOI 10.1371/
journal.pone.0209910.

Santiago-Moreno J., Castaño C., Toledano-Díaz A., Coloma M.A.,
López-Sebastián A., Prieto M.T., Campo J.L. Semen cryopreservation
for the creation of a Spanish poultry breeds cryobank: optimization
of freezing rate and equilibration time. Poult. Sci. 2011;
90(9):2047-2053.

Seigneurin F., Blesbois E. Update on semen cryopreservation methods
in poultry species. In: Proc. of the XIIIth Eur. Poultry Conf.
2010;172. Available at: https://www.visitvalencia.com/sites/default/
files/pdfs/valencia_convention_bureau/candidaturas/epc2022valen
ciabid.pdf

Shahba M., El-Sheshtawy R., El-Azab A., Abdel-Ghaffar A., Ziada M.,
Zaky A. The effect of freeze-drying media and storage temperature
on ultrastructure and DNA of freeze-dried buffalo bull spermatozoa.
Asian Pac. J. Reprod. 2016;5(6):524-535. DOI 10.1016/j.apjr.
2016.11.002.

Shahverdi A., Sharafi V., Gourabi H., Yekta A.A., Esmaeili V., Sharbatoghli
M., Janzamin E., Hajnasrollahi M., Mostafayi F. Fertility
and flow cytometric evaluations of frozen-thawed rooster semen in
cryopreservation medium containing low-density lipoprotein. Theriogenology.
2015;83:78-85. DOI 10.1016/j.theriogenology.2014.
07.044.

Silversides F.G., Robertson M., Liu J. Cryoconservation of avian gonads
in Canada. Poult. Sci. 2013;92(10):2613-2617.

Silyukova Y., Pleshanov N., Stanishevskaya O. The influence membranes
damage andactivity of roosters’ sperm on the fertilization
of eggs when using cured cryopreserved sperm. Reprod. Domest.
Anim. 2019;54(3):101.

Song X.Y., Silversides F. Offspring produced from orthotopic transplantation
of chicken ovaries. Poult. Sci. 2007;86:107-111. DOI
10.1093/ps/86.1.107.

Stanishevskaya O., Pleshanov N. Cryotolerance of cocks’ sperm depending
on their breed and individual properties. Anim. Reprod. Sci.
2018a;194:e1-e27. DOI 10.1016/j.anireprosci.2018.04.031.

Stanishevskaya O., Pleshanov N. Evaluation of the cocks spermatozoa
membranes’ damaging during cryopreservation with use of Sperm
VitalStain colorant. Reprod. Domest. Anim. 2018b;53(2):183.

Stanishevskaya O., Pleshanov N. Livability of chicken embryos, obtained
after insemination by frozen/thawed sperm, depending on
storage duration of incubation eggs. Reprod. Domest. Anim. 2018c;
53(2):199.

Svoradová A., Kuželová L., Vašíček J., Baláži A., Hanusová E.,
Chrenek P. In vitro effect of various cryoprotectants on the semen
quality of endangered Oravka chicken. Zygote. 2017;26(01):33-39.
DOI 10.1017/s0967199417000685.

Svoradová A., Kuželová L., Vašíček J., Olexíková L., Baláži A.,
Kulíková B., Hrnčár C., Ostro A., Bednarczyk M., Chrenek P. The
assessment of cryopreservation on the quality of endangered Oravka
rooster spermatozoa using casa and cytometry. CryoLetters. 2018;
39(6):359-365.

Thélie A., Bailliard A., Seigneurin F., Zerjal T., Tixier-Boichard M.,
Blesbois E. Chicken semen cryopreservation and use for the restoration
of rare genetic resources. Poult. Sci. 2019;98(1):447-455. DOI
10.3382/ps/pey360.

Thieu Ngoc Lan Phuong, Váradi E., Vegi B., Liptói K., Barna J. Comparison
between low/programmable freezing and fast freezing protocols
of Hungarian guinea fowl semen. Athens J. Nat. Formal Sci.
2014;1(3):175-183. DOI 10.13140/2.1.2727.2322.

Tonus C., Cloquette K., Ectors F., Piret J., Gillet L., Antoine N., Desmecht
D., Vanderplasschen A., Waroux O., Grobet L. Long term-cultured
and cryopreserved primordial germ cells from various chicken
breeds retain high proliferative potential and gonadal colonisation
competency. Reprod. Fertil. Dev. 2016;28:628-639.

Tonus C., Connan D., Waroux O., Vandenhove B., Wayet J., Gillet L.,
Desmecht D., Antoine N., Ectors F.J., Grobet L. Cryopreservation
of chicken primordial germ cells by vitrification and slow freezing:
a comparative study. Theriogenology. 2017;88:197-206. DOI
10.1016/j.theriogenology.2016.09.022.

Tselyutin K.V., Tour B.K. Artificial Insemination and Cryopreservation
of Sperm (Roosters, Turkeys, Gander, Drakes). St. Peterburg, 2013.
(in Russian)

Valcarce D.G., Carton-Garcia F., Riesco M.F., Herraez M.P., Robles
V.
Analysis of DNA damage after human sperm cryopreservation in
genes crucial for fertilization and early embryo development. Andrology.
2013;1:723-730. DOI 10.1111/j.2047-2927.2013.00116.x.

van der Lavoir M., Diamond J., Leighton P., Mather-Love C., Heyer
B.,
Bradshaw R., Kerchner A., Hooi L., Gessaro T., Swanberg S., Delany
M., Etches R. Germline transmission of genetically modified
primordial germ cells. Nature. 2006;441(7094):766-769.

Varadi E., Vegi B., Liptoi K., Barna J. Methods for cryopreservation
of Guinea fowl sperm. PLoS One. 2013;8(4):e62759. DOI 10.1371/
journal.pone.0062759.

Vilagran I., Castillo-Martín M., Prieto-Martínez N., Bonet S., Yeste M.
Triosephosphate isomerase (TPI) and epididymal secretory glutathione
peroxidase (GPX5) are markers for boar sperm quality.
Anim. Reprod. Sci. 2016;165:22-30. DOI 10.1016/j.anireprosci.
2015.12.001.

Vilagran I., Yeste M., Sancho S., Castillo J., Oliva R., Bonet S. Comparative analysis of boar seminal plasma proteome from different
freezability ejaculates and identification of Fibronectin 1 as sperm
freezability marker. Andrology. 2015;3(2):345-356. DOI 10.1111/
andr.12009.

Wakayama S., Kamada Y., Yamanaka K., Kohda T., Suzuki H., Shimazu T., Tada M.N., Osada I., Nagamatsu A., Kamimura S., Nagatomo H., Mizutani E., Ishino F., Yano S., Wakayama T. Healthy
offspring from freeze-dried mouse spermatozoa held on the International Space Station for 9 months. Proc. Natl. Acad. Sci. USA. 2017;
14(23):5988-5993. DOI 10.1073/pnas.1701425114.

Wang S., Wang W., Xu Y., Tang M., Fang J., Sun H., Sun Y., Gu M.,
Liu Z., Zhang Z., Lin F., Wu T., Song N., Wang Z., Zhang W., Yin C.
Proteomic characteristics of human sperm cryopreservation. Proteomics. 2014;14:298-310. DOI 10.1002/pmic.201300225.

Watson P.F. The causes of reduced fertility with cryopreserved semen.
Anim. Reprod. Sci. 2000;60-61:481-492.

Wildt D. Genome resource banking for wildlife research, management, and conservation. ILAR J. 2000;41(4):228-234. DOI 10.1093/
ilar.41.4.228.

Woelders H. Animal genetic resources conservation in the Netherlands
and Europe: poultry perspective. Poult. Sci. 2006;85:216-222.

Wu Y., Guo L., Liu Z., Wei H., Zhou Y., Tan J., Peng J. Microelements in seminal and serum plasma are associated with fresh semen
quality in Yorkshire boars. Theriogenology. 2019;132:88-94. DOI
10.1016/j.theriogenology.2019.04.002.

